# Proximity‐Ligation Metagenomic Sequence Analysis Reveals That the Antibiotic Resistome Makes Significant Transitions During Municipal Wastewater Treatment

**DOI:** 10.1111/1462-2920.70036

**Published:** 2025-01-10

**Authors:** Cassandra B. McCorison, Taegyu Kim, Justin J. Donato, Timothy M. LaPara

**Affiliations:** ^1^ Department of Chemistry University of St. Thomas St. Paul Minnesota USA; ^2^ Department of Genetics and Genomic Sciences Icahn School of Medicine at Mount Sinai New York New York USA; ^3^ Department of Civil, Environmental, and Geo‐Engineering University of Minnesota Minneapolis Minnesota USA

**Keywords:** bioinformatics, community genomics, comparative genomics, environmental genomics, functional genomics, genomics, metagenomics, microbial communities, new tools, technological developments

## Abstract

Shotgun and proximity‐ligation metagenomic sequencing were used to generate thousands of metagenome assembled genomes (MAGs) from the untreated wastewater, activated sludge bioreactors, and anaerobic digesters from two full‐scale municipal wastewater treatment facilities. Analysis of the antibiotic resistance genes (ARGs) in the pool of contigs from the shotgun metagenomic sequences revealed significantly different relative abundances and types of ARGs in the untreated wastewaster compared to the activated sludge bioreactors or the anaerobic digesters (*p <* 0.05). In contrast, these results were statistically similar when comparing the ARGs in the pool of MAGs, suggesting that proximity‐ligation metagenomic sequencing is particularly useful for pairing ARGs with their hosts but less adept at discerning quantitative differences in ARG types and relative abundances. For example, numerous MAGs of the genera *Acinetobacter, Enterococcus, Klebsiella* and *Pseudomonas* were identified in the untreated wastewater, many of which harboured plasmid‐borne and/or chromosomal‐borne ARGs; none of these MAGs, however, were detected in the activated sludge bioreactors or anaerobic digesters. In conclusion, this research demonstrates that the antibiotic resistome undergoes significant transitions in both the relative abundance and the host organisms during the municipal wastewater treatment process.

## Graphical abstract

Shotgun and proximity‐ligation metagenomic DNA sequence analysis revealed a significant transition in the antibiotic resistome during municipal wastewater treatment. Antibiotic resistance genes underwent significant changes in both the relative abundance and the host organisms (metagenome assembled genomes) from the untreated wastewater to the activated sludge bioreactors to the anaerobic digesters.

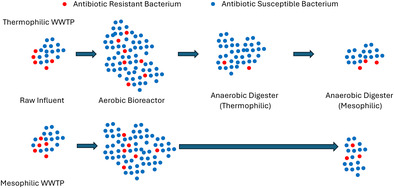

## 1 Introduction

Antibiotics and antibacterials are natural and synthetic compounds, respectively, that specifically inhibit and/or inactivate microorganisms (Kohanski, Dwyer, and Collins 2010; Smith et al. 2023). Antibiotics and antibacterials (henceforth referred to simply as ‘antibiotics’) are invaluable for protecting and maintaining public health because these medicines can be used to treat numerous bacterial infections without directly affecting the infected individuals (Kohanski, Dwyer, and Collins 2010; Smith et al. 2023). Unfortunately, bacterial resistance to antibiotics is increasing. A recent report suggested that there are nearly 3 million antibiotic‐resistant infections leading to more than 35,000 deaths in the United States each year (CDC 2019). Another report predicted that antibiotic resistance would contribute to almost 40 million deaths by 2050 (GBD 2021 Antimicrobial Resistance Collaborators 2024).

To prevent and to slow the spread of antibiotic resistance, a multi‐pronged approach has been recommended by the US Centers for Disease Control and Prevention, including initiatives to prevent device‐ and procedure‐related infections, to improve hygiene to limit the spread of pathogens between health care facilities, to limit inappropriate antibiotic use, to encourage more widespread use of vaccines and to implement safer food handling practices (CDC 2019). In addition, a One Health approach to antibiotic resistance is gaining traction (Adisasmito et al. 2022; Aslam et al. 2021), such that environmental reservoirs of antibiotic resistance are being identified and solutions to resolve these reservoirs are being proposed (Crofts, Gasparrini, and Dantas 2017; Ding et al. 2023; Marti, Variatza, and Balcazar 2014; Pruden et al. 2006). Of particular interest is untreated municipal wastewater, which is known to contain high concentrations and diverse populations of antibiotic resistance genes (ARGs) and antibiotic resistant bacteria (ARB) (Buelow et al. 2018; Che et al. 2019; Munk et al. 2022; Perez‐Bou et al. 2023). The focus on municipal wastewater as a pertinent reservoir of antibiotic resistance is particularly attractive because many parts of the world already have already implemented substantial infrastructure to collect and to treat untreated wastewater (Tchobanoglous et al. 2014), such that subtle tweaks to the existing installations could presumably achieve substantial reductions in this reservoir of antibiotic resistance.

The historical goal of municipal wastewater treatment has been to provide sufficient treatment so that wastewater can be released without adverse impacts to the environment (Tchobanoglous et al. 2014). Each municipal wastewater treatment facility is unique, involving numerous physical, chemical and biological unit operations. Prior research, therefore, has explored whether filtration (Keenum et al. 2024; LaPara et al. 2011; Li et al. 2023) and effluent disinfection (Lin et al. 2024) can limit the release of ARGs and ARB with the treated wastewater. In contrast, prior research has suggested that approximately 95% of the ARGs in the untreated wastewater are diverted to the sewage sludge (Munir, Wong, and Xagoraraki 2011), such that numerous researchers have investigated the ability of various sewage sludge treatment technologies to reduce ARG and ARB levels (Cui et al. 2022; Wang et al. 2023, 2024). Of particular interest is the anaerobic digestion of sewage sludge, which is used to treat more sewage sludge than any other technology in the United States (Beecher et al. 2007). Some researchers have suggested that high‐temperature anaerobic digestion (a.k.a., ‘thermophilic anaerobic digestion’, typically operated at temperatures > 50°C) achieves better reductions in ARGs than conventional anaerobic digestion (a.k.a., ‘mesophilic anaerobic digestion’, typically operated at temperatures between 35°C and 37°C) (Burch, Sadowsky, and LaPara 2016; Diehl and LaPara 2010; Ghosh, Ramsden, and LaPara 2009; Zhang et al. 2021), although other researchers have observed similar ARG removal efficiencies independent of the temperature at which anaerobic digestion is performed (Huang et al. 2019; Ma et al. 2011; Zhang, Yang, and Pruden 2015).

In this study, we compared the ARG levels and composition in the untreated wastewater, the activated sludge bioreactors and the anaerobic digesters from two different full‐scale municipal wastewater treatment facilities. These two wastewater treatment facilities were of similar design, except one used a conventional anaerobic digester (operating temperature = 37°C) to treat its sewage sludge whereas the other facility used a two‐stage anaerobic digester in which the first stage was operated at elevated temperature (55°C) and the second stage was operated at conventional temperature (37°C). Samples were analysed by shotgun and proximity‐ligation metagenomic sequencing, which enables more accurate and extensive compiling of metagenome assembled genomes (MAGs) compared to shotgun metagenomic sequencing alone. This improvement is achieved by using formaldehyde to covalently bond DNA molecules in close proximity to each other within the original sample, allowing chromosomal and plasmid sequences obtained from within a single cell to be more accurately linked to each other (Stalder et al. 2019). This approach allowed us to determine the relative abundance of hundreds of different ARGs (similar to prior studies using shotgun metagenomics) but then also pairing these ARGs with their host organism, including whether these ARGs were located on a plasmid or chromosome.

## 2 Experimental Procedures

### 2.1 Site Descriptions and Sample Collection

Samples were collected from the two wastewater treatment facilities in the upper Midwest of the United States on three different dates approximately 2 months apart. Facility M uses a conventional process (bar rack, grit removal, primary clarifier, aeration basin, secondary clarifier, effluent disinfection) to treat approximately 40,000 m^3^ of wastewater each day. Facility T uses a similar process to treat approximately 11,000 m^3^ of wastewater each day. Both facilities generate a mixture of primary and secondary solids as sewage sludge. Facility M uses a single‐stage anaerobic digester operated at 37°C to treat its sewage sludge. In contrast, Facility T uses a two‐stage anaerobic digester system to treat its sewage sludge; the first stage is operated at 55°C and then second stage is operated at 37°C.

All samples were collected in sterile containers and immediately transported to the University of St. Thomas on ice. Untreated wastewater samples (10 mL) were collected following grit removal but prior to treatment by the primary clarifiers. Activated sludge (1 mL) and anaerobic digester (0.1 mL) samples were directly collected from the aeration basins and anaerobic digesters, respectively. All samples were concentrated by centrifugation (3 min; 5000× *g*), the supernatant was removed, and the cell pellet was frozen at −20°C. Samples were shipped overnight to Phase Genomics on dry ice.

### 2.2 Metagenomic Sequencing

A Hi‐C library was created for each sample using the Phase Genomics ProxiMeta Hi‐C v4.0 Kit (Lieberman‐Aiden et al. 2009). Briefly, intact cells were crosslinked using a formaldehyde solution, simultaneously digested using the Sau3AI and MlucI restriction enzymes, and proximity‐ligated with biotinylated nucleotides to create chimeric molecules composed of fragments from different regions of genomes that were physically proximal in vivo. Proximity‐ligated DNA molecules were concentrated using streptavidin beads and processed into an Illumina‐compatible sequencing library. Separately, using an aliquot of the original sample, DNA was extracted using a ZYMObiomics DNA miniprep kit (Zymo Research, Irvine, California, USA), from which a metagenomic shotgun library was prepared using ProxiMeta library preparation reagents. Sequencing was performed using an Illumina NovaSeq generating PE150 read pairs for both Hi‐C and shotgun libraries.

### 2.3 Metagenomic Sequence Analysis

Shotgun reads were processes using fastp (version 0.20.1) (Chen et al. 2018) with the default parameters for adapter trimming and read quality filtering, followed by assembly using MEGAHIT (version 1.2.9) using default parameters (Li et al. 2015, 2016). Hi‐C reads were then aligned to the metagenomic assembly according to the manufacturer's recommendations using BWA‐MEM (version 0.7.17) using the ‐5SP option (Li and Durbin 2010). PCR duplicates were flagged and excluded from analysis using SAMBLASTER (version 0.1.24) (Faust and Hall 2014). Alignments were then filtered with SAMtools using the ‐F 2304 flag to remove non‐primary and secondary alignments (Li et al. 2009). The metagenomic assembly was binned using the ProxiMeta platform, which uses a graph‐based clustering algorithm that combines shotgun and Hi‐C evidence to generate high quality MAGs. (Press et al. 2017; Stewart et al. 2018).

Genome clusters were assessed for quality using CheckM (version 1.1.3) (Parks et al. 2015). Preliminary taxonomic classification was performed using Mash (version 2.1) (Ondov et al. 2016). Further taxonomic classification was performed using the Genome Taxonomy Database Toolkit (GTDB‐Tk, v2.4.0) and database v220 using the default parameters (Chaumeil et al. 2019, 2022; Parks et al. 2018, 2020, 2022; Rinke et al. 2021).

Viral contigs were identified using VIBRANT (version 1.2.1) with default settings (Kieft, Zhou, and Anantharaman 2020). Putative viral contigs with bacterial and viral sequences present were annotated as prophages if 50% or more of the contig length was annotated as viral, otherwise they were annotated as bacterial. Plasmid contigs were identified by blastn in the assembly to a custom version of the PLSDB database (version 2020_06_29) (Galata et al. 2020). Plasmid hit quality was determined based solely on the alignment to the best hit in the database, with secondary hits not being considered. The quality score was based on the percentage of the reference plasmid covered by the query contig. Several thresholds were applied to ensure high‐quality plasmid identification, including percent identity (alignments > 90% identity were considered significant), alignment length (a minimum length of 100 bp was used to filter out short, spurious matches), and coverage (contigs were classified as a plasmid if at least 50% of its length was aligned to a reference plasmid and if at least 50% of the reference plasmid was covered by the contig). Hi‐C data was also used to bin plasmid contigs into ‘metagenome assembled plasmids’ using the same logic that was used for binning plasmid contigs (Uritskiy et al. 2021). The quality of viral contigs was assessed using CheckV (version 1.0.1) (Nayfach et al. 2021). Both viral and plasmid host assignment was carried out using the ProxiPhage host attribution tool (Uritskiy et al. 2021). Long‐range Hi‐C linkages between viral/plasmid contigs and their prokaryotic host genomes were analysed to assign likely hosts.

ARGs were annotated from the metagenomic assembly using AMRFinderPlus (version 3.10.5) with the—plus option enabled and default parameters (Feldgarden et al. 2021). Annotated ARGs were cross‐referenced with MAG taxonomic assignments and the host‐viral and host‐plasmid association matrices generated by ProxiMeta to determine the origin of ARGs (i.e., chromosomal, viral, or plasmid). A binary matrix was created to track the presence of ARGs in each MAG and classify their origin accordingly. The relative abundance of ARGs was computed by dividing the number of annotated ARGs by the total quantity of DNA sequence information (typically reported as the number of ARGs per 10^8^ base pairs).

### 2.4 Statistical Analysis

All statistical analyses were performed in R. Tukey and T‐test statistical analyses were performed using the stats package. Bray–Curtis dissimilarity and the analysis of similarities (anosim) were performed using the vegan package (Oksanen et al. 2024). Principal coordinates analysis was performed using the ape package (Paradis and Schliep 2019). Graphs were generated using the Tidyverse, ggplot2 and pheatmap packages (Wickham 2009; Wickham et al. 2019).

## 3 Results

Direct shotgun metagenomic sequences (1.0 × 10^10^ total reads) and proximity‐ligation sequences (4.5 × 10^9^ total reads) were obtained, which were then subsequently used to generate profiles of contigs and MAGs from each sample (Table 1). More than 5000 MAGs were generated, of which 2257 qualified as ‘medium quality’ (at least 50% estimated genome completion and a marker gene overrepresentation of < 10%) per the standards set by the Genome Standards Consortium (Bowers et al. 2017). Henceforth, these medium quality MAGs are referred to as ‘filtered MAGs’. Although numerous MAGs had an estimate genome completeness > 90% and an marker gene overrepresentation of < 5%, none of these MAGs qualified as ‘high quality’ because of an insufficient number of ribosomal and transfer RNA genes.

**TABLE 1:** Summary statistics of proximity‐ligation metagenomic sequencing from two full‐scale municipal wastewater treatment facilities.

	Location and timepoint	Shotgun reads^a^	Hi‐C reads^a^	Contigs	Contig assembled length (bp)	MAGs	MAG assembled length (bp)	Filtered MAGs^b^	Filtered MAG assembled length (bp)
Facility M	Raw influent 1	7.0 × 10^8^	2.0 × 10^8^	650,940	1.7 × 10^9^	287	5.6 × 10^8^	101	4.3 × 10^8^
	Raw influent 2	4.6 × 10^8^	2.0 × 10^8^	757,081	2.0 × 10^9^	394	5.5 × 10^8^	164	3.8 × 10^8^
	Raw influent 3	3.5 × 10^8^	2.6 × 10^8^	525,522	1.3 × 10^9^	241	5.6 × 10^8^	83	4.2 × 10^8^
	Activated sludge 1	7.0 × 10^8^	1.7 × 10^8^	810,160	2.7 × 10^9^	332	2.8 × 10^8^	131	1.8 × 10^8^
	Activated sludge 2	4.6 × 10^8^	1.3 × 10^8^	707,402	2.2 × 10^9^	235	6.3 × 10^8^	93	4.6 × 10^8^
	Activated sludge 3	4.4 × 10^8^	1.6 × 10^8^	642,852	2.0 × 10^9^	264	6.5 × 10^8^	105	5.1 × 10^8^
	Mesophilic digester 1	4.0 × 10^8^	1.3 × 10^8^	525,080	1.6 × 10^9^	120	3.5 × 10^8^	44	2.4 × 10^8^
	Mesophilic digester 2	5.0 × 10^8^	2.1 × 10^8^	613,423	1.9 × 10^9^	352	7.1 × 10^8^	161	5.3 × 10^8^
	Mesophilic digester 3	5.7 × 10^8^	1.7 × 10^8^	688,403	2.1 × 10^9^	431	1.7 × 10^8^	198	1.2 × 10^8^
Facility T	Raw influent 1	2.6 × 10^8^	1.3 × 10^8^	603,885	1.5 × 10^9^	187	5.1 × 10^8^	64	3.1 × 10^8^
	Raw influent 2	1.4 × 10^8^	1.1 × 10^8^	214,663	5.1 × 10^8^	374	6.5 × 10^8^	122	4.8 × 10^8^
	Raw influent 3	5.4 × 10^8^	1.2 × 10^8^	682,080	1.7 × 10^9^	111	4.7 × 10^8^	51	3.5 × 10^8^
	Activated sludge 1	6.7 × 10^8^	2.2 × 10^8^	569,372	1.7 × 10^9^	329	2.0 × 10^8^	134	1.4 × 10^8^
	Activated sludge 2	6.8 × 10^8^	2.5 × 10^8^	662,058	1.9 × 10^9^	389	2.5 × 10^8^	138	1.7 × 10^8^
	Activated sludge 3	6.2 × 10^8^	1.9 × 10^8^	614,028	1.8 × 10^9^	346	4.8 × 10^8^	127	3.4 × 10^8^
	Thermophilic digester 1	3.8 × 10^8^	5.4 × 10^8^	126,791	4.2 × 10^8^	122	7.2 × 10^8^	60	4.9 × 10^8^
	Thermophilic digester 2	3.4 × 10^8^	1.7 × 10^8^	120,088	3.6 × 10^8^	103	1.8 × 10^8^	48	1.3 × 10^8^
	Thermophilic digester 3	2.6 × 10^8^	1.7 × 10^8^	152,823	4.8 × 10^8^	136	4.7 × 10^8^	75	3.8 × 10^8^
	Mesophilic digester 1	5.7 × 10^8^	3.6 × 10^8^	279,832	8.5 × 10^8^	227	2.5 × 10^8^	118	1.6 × 10^8^
	Mesophilic digester 2	7.6 × 10^8^	3.2 × 10^8^	492,630	1.5 × 10^9^	290	8.4 × 10^8^	117	5.5 × 10^8^
	Mesophilic digester 3	5.5 × 10^8^	3.0 × 10^8^	288,854	8.9 × 10^8^	217	2.6 × 10^8^	123	2.1 × 10^8^

^a^ Paired end, includes both read 1 and read 2.

^b^ The MAGs with ≥ 50% estimated completion and < 10% of marker genes overrepresented.

### 3.1 Quantitative Comparisons of the ARGs in Contigs, MAGs and Filtered MAGs

**FIGURE 1:**
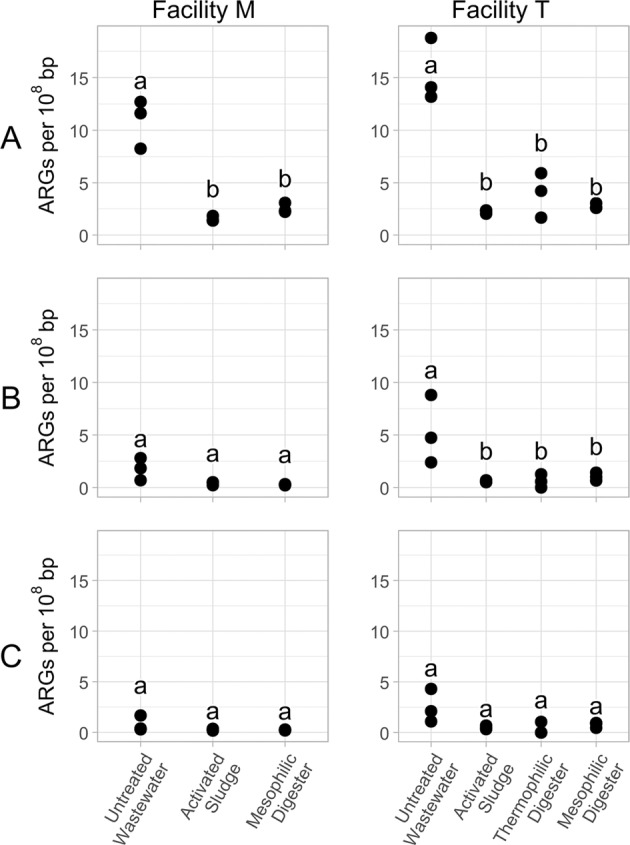
The relative abundance of antibiotic resistance genes (ARGs) in the untreated wastewater, activated sludge bioreactors and anaerobic digesters from two full‐scale municipal wastewater treatment facilities. The relative abundance of ARGs was computed from (A) metagenomic sequences assembled into contigs, (B) metagenome assembled genomes (MAGs) assembled by proximity ligation sequencing, and (C) the pool of ‘filtered’ MAGs, which excluded MAGs for insufficient completeness (< 50%) or for excessively high marker gene overrepresentation rate (> 10%). Letters indicate significance groups (*p* < 0.05).

The quantities of ARGs in the contigs, MAGs and filtered MAGs were compared in the untreated wastewater, activated sludge bioreactors, mesophilic digesters and thermophilic digester (Figure 1). The relative abundance of ARGs in the contigs was significantly greater (*p* < 0.001) in the untreated wastewater compared to the activated sludge, the mesophilic digesters and the thermophilic digester at both Facility M and Facility T. In contrast, at Facility M, the relative abundance of ARGs in the pool of MAGs were higher in the untreated wastewater compared to the activated sludge and the mesophilic digester, albeit at a threshold that was not statistically significant (*p* = 0.06 and *p* = 0.07, respectively). The results at Facility T were similar, although statistically significant (*p* = 0.03), when comparing the untreated influent with either the activated sludge or the thermophilic digester; curiously, the relative abundance of ARGs in the untreated influent was not statistically significant compared to the mesophilic digester at Facility T (*p* = 0.054). In the filtered MAGs, there were no statistically significant differences when comparing the relative abundance of ARGs in the untreated influent, activated sludge, thermophilic digester, and/or mesophilic digesters. However, at Facility T, the relative abundance of ARGs in the untreated wastewater was higher than in the activated sludge or the thermophilic digester, albeit at a level that was not statistically significance (*p* = 0.09 and *p* = 0.07, respectively).

**FIGURE 2:**
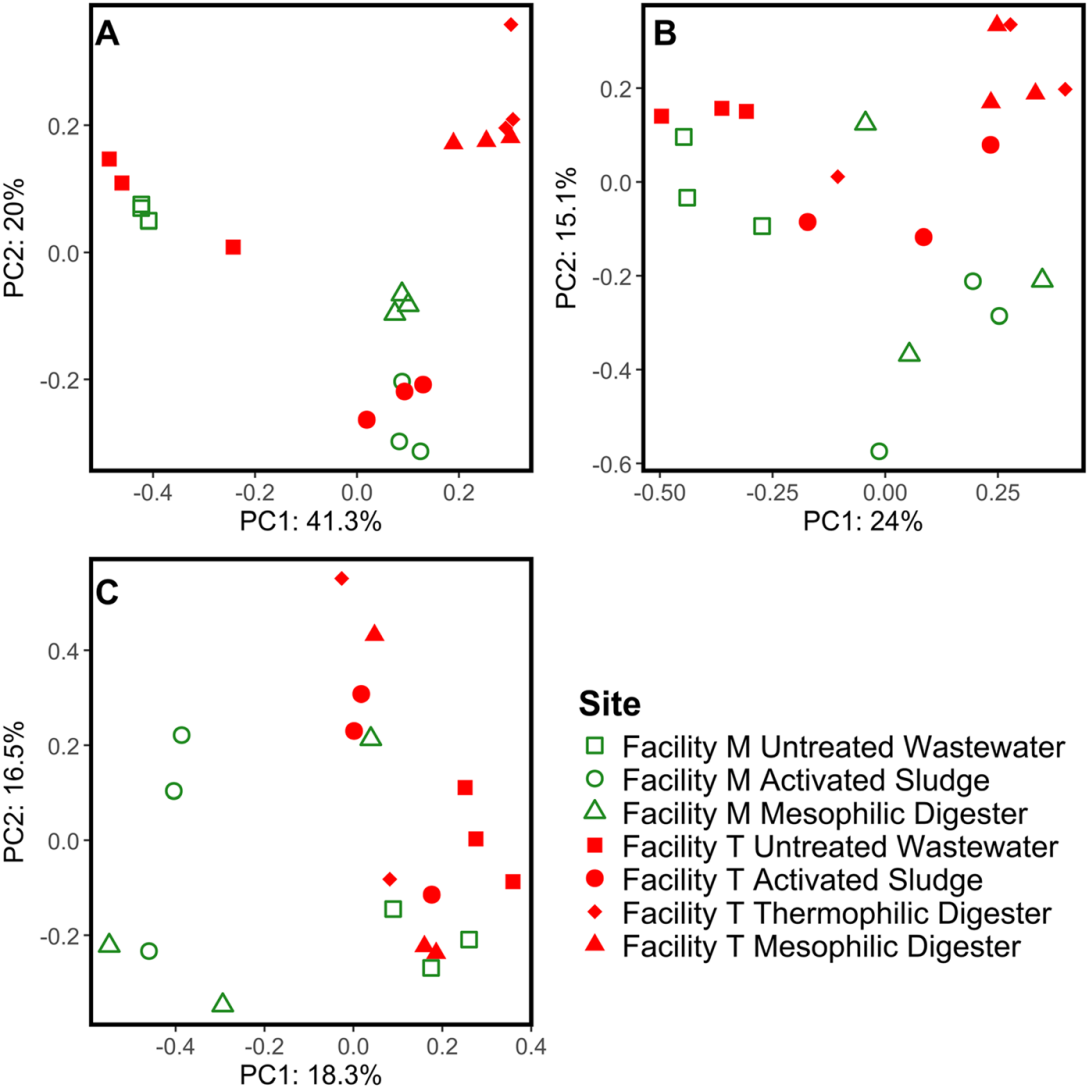
Principal component analysis (PCoA) of the Bray–Curtis Dissimilarity matrix of different antibiotic resistance genes (ARGs) in the untreated wastewater, activated sludge bioreactors, and anaerobic digesters from two full‐scale municipal wastewater treatment facilities. The ordination plots were computed from (A) metagenomic sequences assembled into contigs, (B) metagenome assembled genomes (MAGs) assembled by proximity ligation sequencing, and (C) the pool of ‘filtered’ MAGs, which excluded MAGs for insufficient completeness (< 50%) or for excessively high marker gene overrepresentation rate (> 10%).

The composition of ARGs in the contigs, MAGs and filtered MAGs were compared by principal coordinates analysis of the Bray–Curtis dissimilarities of the quantities and types of ARGs in each sample (Figure 2). Qualitatively, the ARG profiles obtained from the contigs demonstrated that samples collected on different dates but from the same location (untreated wastewater, activated sludge, thermophilic digester and mesophilic digesters) at the same facility clustered closely with the samples collected from the same facility and location (Figure 2A), although these differences were not statistically significant (*p* > 0.1) Similarly, the ARG profiles from the untreated wastewater and activated sludge samples clustered with the analogous samples collected from the other facility, such that the ARG profiles of the raw wastewater, activated sludge and anaerobic digester from both facilities were significantly different from each other (*p* < 0.05). Alternatively, the ARG profiles from the mesophilic and thermophilic anaerobic digester samples from Facility T were statistically similar to each other (*p* > 0.1) but significantly different from the anaerobic digester samples from Facility M (*p* < 0.05).

The ARG profiles obtained from the MAGs (Figure 2B) and filtered MAGs (Figure 2C) exhibited more variation than the ARG profiles obtained from the contigs (Figure 2A). Although some of the ARG profiles from the same facility and location loosely clustered together (e.g., the untreated wastewater samples from both facilities), the ARG profiles from the raw wastewater, activated sludge, and anaerobic digester samples were not significantly different from each other (*p* > 0.1). Some statistically significant differences in ARG profiles were nonetheless still observed. For example, the ARG profiles from the raw wastewater samples were significantly different from the ARG profiles from the activated sludge and anaerobic digester samples (*p* < 0.05). In addition, the ARG profiles from the anaerobic digesters from Facility T were significantly different from the ARG profiles from the activated sludge samples, although ARG profiles from the activated sludge samples were statistically similar to the ARG profiles from the anaerobic digesters at Facility M (*p* > 0.1).

**FIGURE 3:**
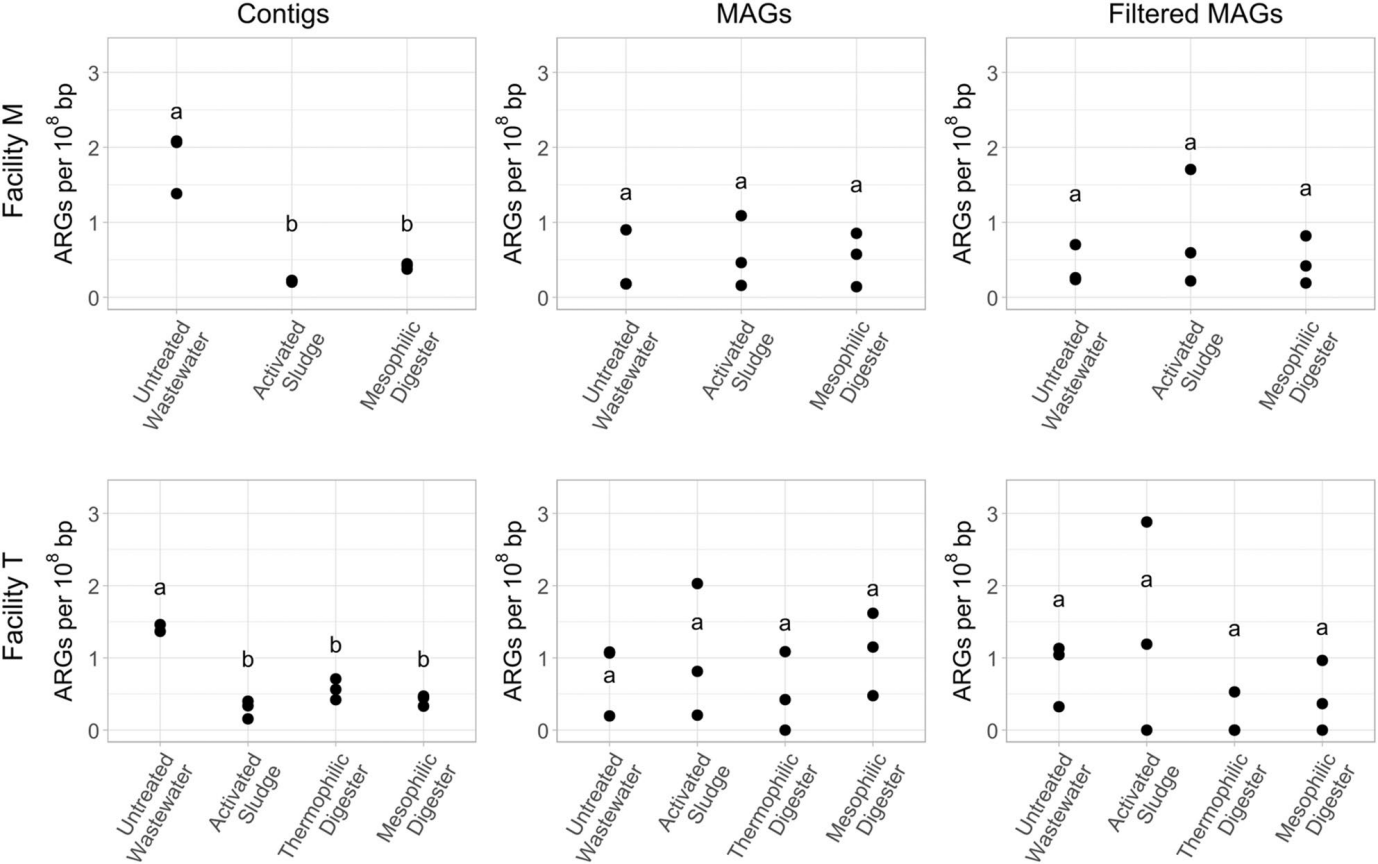
The relative abundance of genes encoding resistance to aminoglycosides in the untreated wastewater, activated sludge bioreactors, and anaerobic digesters from two full‐scale municipal wastewater treatment facilities (Facility M: Top panel; Facility T: Bottom panel). Results were computed from metagenomic sequences directly assembled into contigs, from proximity‐ligation sequence results assembled into metagenome assembled genomes (MAGs), and from the pool of ‘filtered’ MAGs, which excluded MAGs for insufficient completeness (< 50%) or for excessively high marker gene overrepresentation rate (> 10%). Letters indicate significance groups (*p* < 0.05).

The four most prominent classes of ARGs in the contigs, MAGs and filtered MAGs encoded resistance against aminoglycosides, β‐lactams, macrolides and tetracyclines. In both facilities, the number of genes within the aminoglycosides and β‐lactams found within the contig dataset significantly (*p* < 0.05) decreased from the untreated wastewater samples compared to the activated sludge, thermophilic digester and mesophilic digesters (Figures 3 and S1). Similar reductions in the relative abundance of macrolides and tetracyclines were also observed in the contigs, although the reduction from untreated wastewater to thermophilic digester was not statistically significant (*p* = 0.2 and *p* = 0.1, respectively) (Figures S2 and S3). In contrast, no statistically significant reductions were observed in the MAGs and filtered MAGs in the untreated wastewater compared to the activated sludge, thermophilic digester and mesophilic digesters for any of these four gene classes (Figure 3 and Figure S1).

**FIGURE 4:**
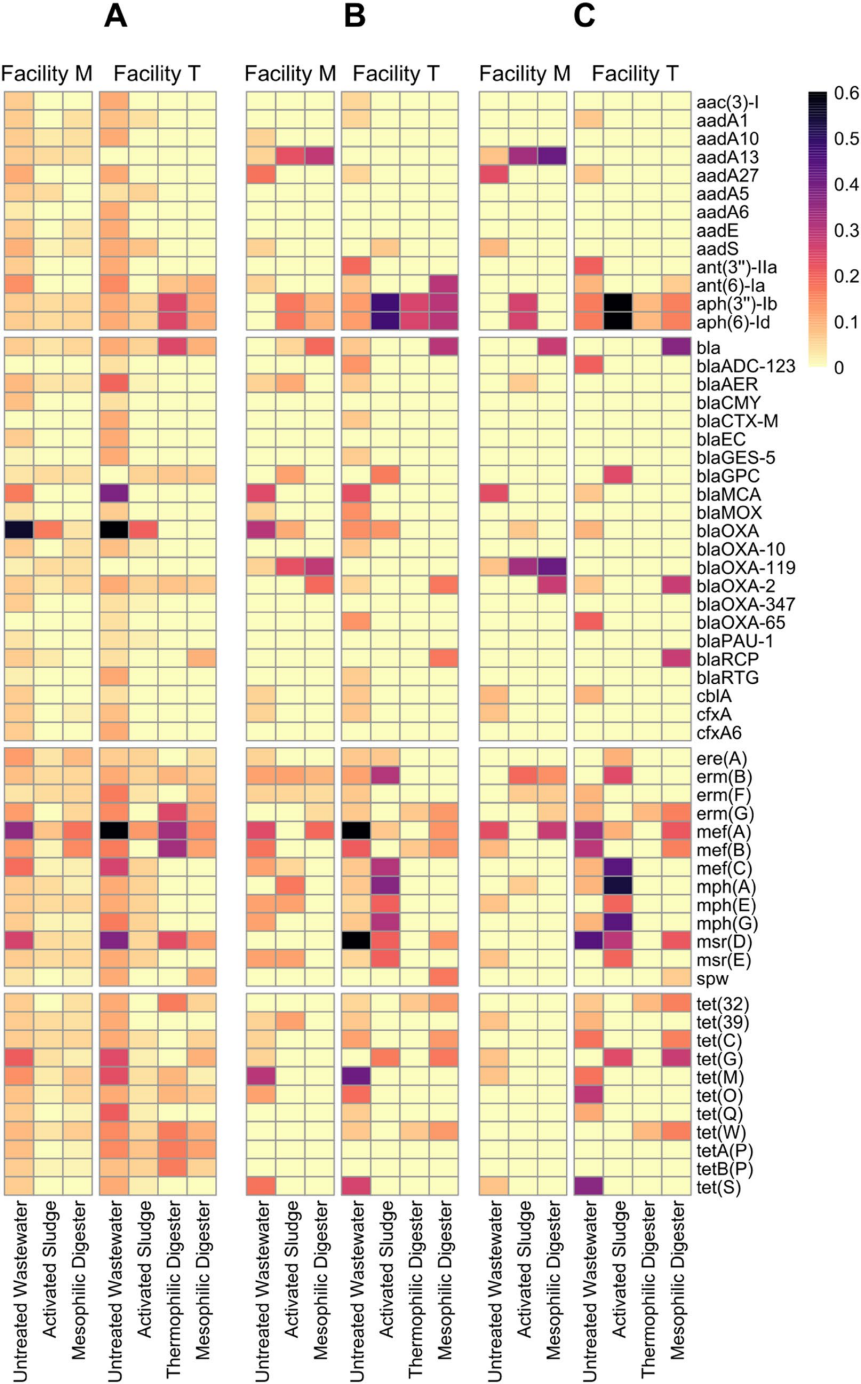
The relative abundance of individual antibiotic resistance genes (ARGs) in the untreated wastewater, activated sludge bioreactors, and anaerobic digesters from two full‐scale municipal wastewater treatment facilities. The ARGs shown in this figure comprise the most abundant 70% of each class of resistance genes in the pool of assembled contigs. Results were obtained from the (A) metagenomic sequences assembled into contigs, (B) metagenome assembled genomes (MAGs) assembled by proximity ligation sequencing, and (C) the pool of ‘filtered’ MAGs, which excluded MAGs for insufficient completeness (< 50%) or for excessively high marker gene overrepresentation rate (> 10%). The scale bar shows the number of ARGs detected per 10^8^ assembled nucleotides.

Individual ARGs were also tracked in the untreated wastewater, activated sludge and anaerobic digester samples from both facilities (Figure 4A). A total of 254 different ARGs were detected in the contigs from the 21 samples. Of these, 73 ARGs were found only once, comprising 4.6% of the total ARGs found within these two facilities. In contrast, three ARGs comprised 11.7% of the total ARGs; these ARGs were *blaOXA*, *mef*(A) and *msr*(D). In Facility M, the untreated wastewater had a greater variety and abundance of ARGs compared to the activated sludge and mesophilic digester samples. In Facility T, the untreated wastewater also had a greater variety and abundance of ARGs compared to the activated sludge and anaerobic digester samples; however, several ARGs were detected in higher abundance in the thermophilic anaerobic digester samples compared to either the untreated wastewater or activated sludge samples.

After assembling the contigs into the pools of MAGs and filtered MAGs, substantially different patterns were observed in the fate of ARGs from the untreated wastewater to the activated sludge and/or anaerobic digesters (Figure 4B,C). Several genes that were found in the pool of contigs in the untreated wastewater were not detected in the pools of MAGs and filtered MAGs. In addition, there were numerous ARGs found in the MAGs and filtered MAGs in the activated sludge and anaerobic digester samples that were not detected in the MAGs and filtered MAGs obtained from the untreated wastewater samples. In fact, a handful of ARGs (e.g., *aph(3″)‐Ib, aph(6)‐Id, mef*(C), *mph*(A), *mph*(G)) were most prominent in the filtered MAGs from the activated sludge samples from Facility T. There were fewer ARGs detected in the filtered MAGs of the thermophilic anaerobic digester; curiously, there were substantially more ARGs in the mesophilic anaerobic digester at Facility T, even though this unit operation is downstream of the thermophilic anaerobic digester.

### 3.2 Pairing ARGs With MAGs

**FIGURE 5:**
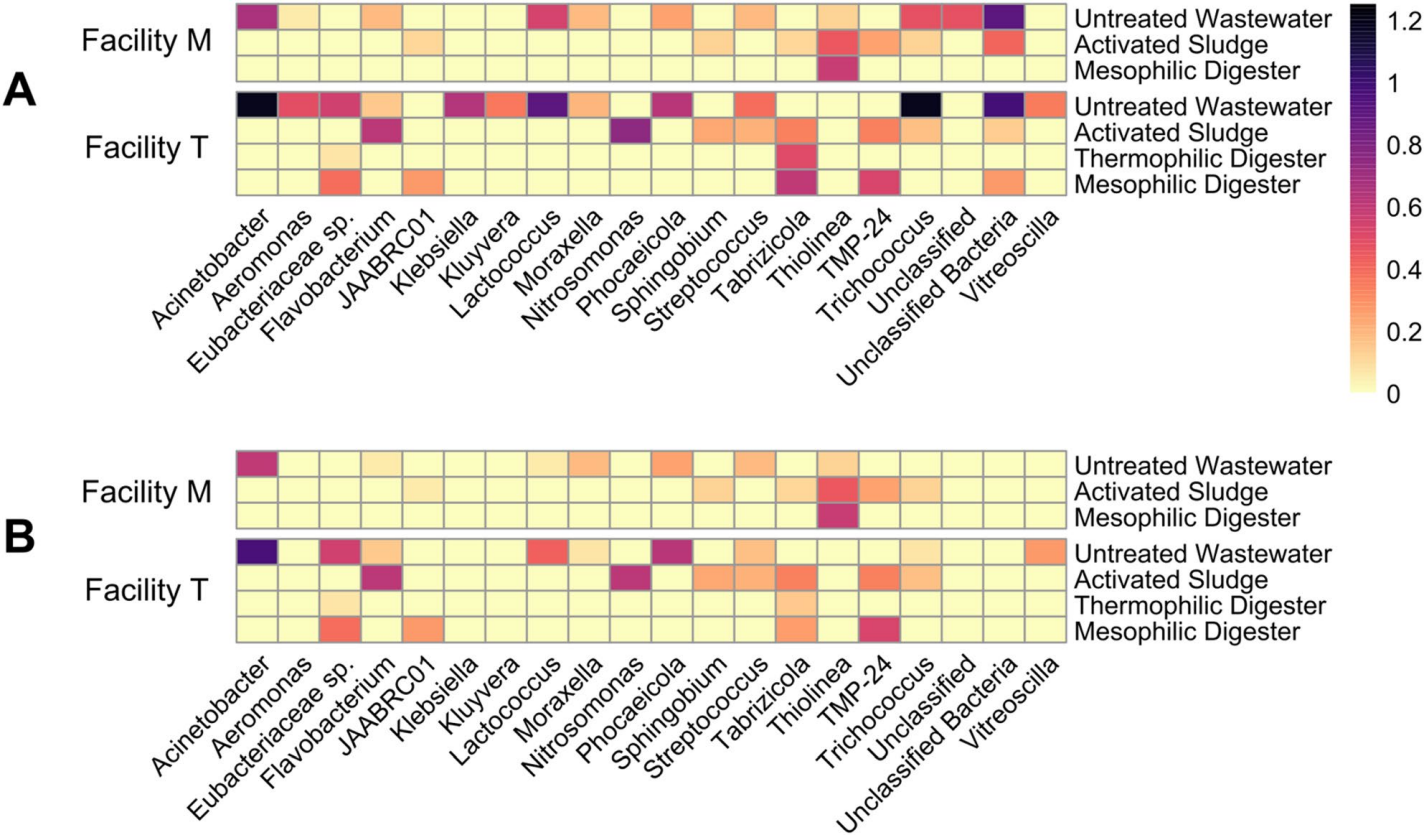
The prevalence of antibiotic resistance genes (ARGs) in metagenome assembled genomes (MAGs) detected in the untreated wastewater, activated sludge bioreactors, and anaerobic digesters from two full‐scale municipal wastewater treatment facilities. The ARGs shown in this figure comprise the most abundant 70% of each class of resistance genes in the pool of assembled contigs. Results are shown from (A) MAGs assembled by proximity ligation sequencing, and (B) the pool of ‘filtered’ MAGs, which excluded MAGs for insufficient completeness (< 50%) or for excessively high marker gene overrepresentation rate (> 10%). The scale bar shows the number of ARGs detected per 10^8^ assembled nucleotides.

Several prominent bacterial genera were identified as hosts for ARGs in the untreated wastewater, activated sludge, and anaerobic digester samples (Figure 5). ARG‐harbouring MAGs of the genera *Acinetobacter, Aeromonas, Klebsiella* and *Streptococcus* were identified in the untreated wastewater at Facility M and/or Facility T, which is of particular concern because these organisms are known human pathogens (De Oliveira et al. 2020). However, in the pool of filtered MAGs, ARG‐harbouring *Aeromonas* spp. and *Klebsiella* spp. were not observed. Otherwise, the profiles of the ARG‐harbouring genera in the activated sludge and anaerobic digesters were similar between the pools of MAGs and filtered MAGs. Several ARG‐harbouring MAGs were detected in the activated sludge samples, but not in the untreated wastewater, including MAGs of the genera *Sphingobium* (Facility M and T), *Tabrizicola* (Facility M and T) and *Nitrosomonas* (Facility T). At Facility M, the most prominent ARG‐harbouring MAGs in the mesophilic anaerobic digester were of the genus *Thiolinea*, which were also found in both the untreated wastewater and activated sludge samples. Similarly, the most prominent ARG‐harbouring MAGs in the thermophilic anaerobic digester at Facility T were of the genus *Tabrizicola*, which was also found in the activated sludge and mesophilic anaerobic digester samples, but not in the untreated wastewater samples. Curiously, several ARG‐harbouring genera (e.g., *Eubacteriaeceae* sp., JAABRC01, *Tabrizicola* and TMP‐24) became more prominent in the mesophilic anaerobic digester samples at Facility T.

**FIGURE 6:**
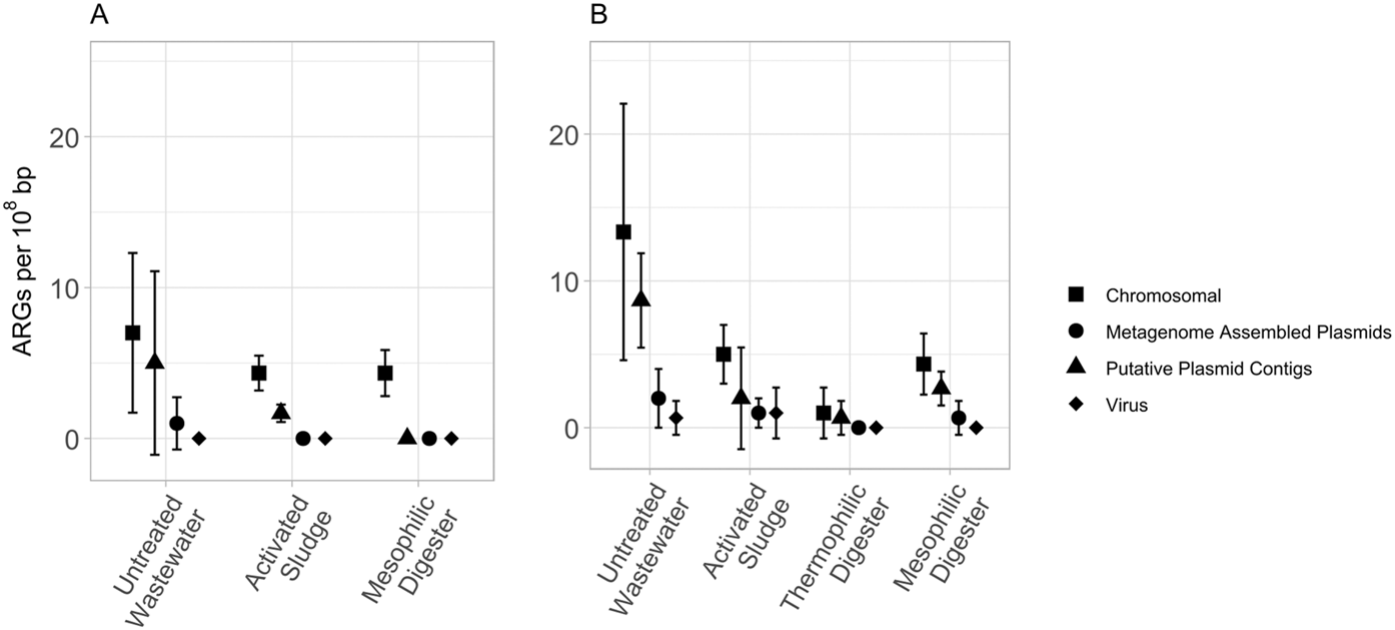
The relative abundance of antibiotic resistance genes (ARGs) associated with chromosomes, metagenome assembled plasmids, putative plasmid contigs, and viruses detected in the filtered metagenome assembled genomes (MAGs) detected in the untreated wastewater, activated sludge bioreactors, and anaerobic digesters from (A) Facility M, and (B) Facility T. Results are shown as the arithmetic mean (± standard deviation; *n =* 3) of the number of ARGs per 10^8^ nucleotides.

Correctly distinguishing plasmids from other mobile genetic elements within metagenomic datasets poses a significant challenge. One advantage of proximity‐ligation sequencing is that putative plasmid contigs can be linked directly to a host to create a metagenome assembled plasmid, which increases the confidence that these putative plasmid identifications are genuine. We therefore compared the relative quantities of ARGs found on chromosomes, metagenome assembled plasmids and putative plasmid contigs (Figure 6). In the untreated wastewater, the frequency of detecting an ARG on a chromosome was statistically similar to the frequency of detecting an ARG on a putative plasmid contig (*p* = 0.9 for Facility M; *p* = 0.6 for Facility T). There was a more substantial difference when comparing the prevalence of ARGs on chromosomes versus those on metagenome assembled plasmids in the untreated wastewater, but these differences were not statistically significant (*p* = 0.3 for Facility M; *p* = 0.10 for Facility T). In contrast, at Facility M, ARGs were significantly more likely to be found on a chromosome than either than metagenome assembled plasmid or putative plasmid contigs in both the activated sludge and mesophilic anaerobic digester samples (*p* < 0.02). However, at Facility T, ARGs were not significantly more likely to be found on a chromosome than either the metagenome assembled plasmids or in the pool of putative plasmid contigs in either the activated sludge or anaerobic digester samples (*p* ≥ 0.2). In addition, proximity‐ligation sequencing can also identify viral DNA within a genome. No ARGs within viral DNA were detected within the filtered MAGs from Facility M; however, small quantities of ARGs with viral DNA were detected at Facility T in the untreated wastewater and activated sludge samples.

The untreated wastewater from both facilities contained organisms of the genera *Enterococcus, Staphylococcus, Klebsiella, Acinetobacter, Pseudomonas* and *Enterobacter* (ESKAPE‐related) that were associated with both metagenome assembled plasmids and putative plasmid contigs (Table 2). In the untreated wastewater samples, several different MAGs identified as *Acinetobacter* had numerous ARGs located on the chromosome, metagenome associated plasmids, and host‐specific putative plasmid contigs. Two different MAGs identified as *Enterococcus*, one each from Facility M and T, were found in the untreated wastewater, each with a single ARG found within the pool of putative plasmid contigs. Three different MAGs identified as *Klebsiella* were detected in the untreated wastewater from Facility T, each containing ARGs on either on the chromosome or the pool of putative plasmid contigs. These *Klebsiella* MAGs were generally of low quality due to a low estimated completion percentage such that they were not included in the pool of filtered MAGs. Curiously, none of the activated sludge or anaerobic digester samples from either facility contained ESKAPE‐related genera.

**TABLE 2:** Antibiotic resistance genes (ARGs) detected within metagenome assembled genomes (MAGs) identified as being ESKAPE genera in samples from the untreated wastewater of two full‐scale municipal wastewater treatment facilities.

	Species^a^	Chromosomal ARGs	ARGs located on metagenome assembled plasmids	ARGs on putative plasmid contigs
Facility M	**Acinetobacter sp**.	—	—	—
	*Acinetobacter* sp.	—	—	—
	**Acinetobacter sp**.	*bla*_MCA_, *mph*(E), *msr*(E)	*aadA27*, *tet*(39)	*bla*_OXA‐58_, *aadA27*, *bla*_MCA_
	**Acinetobacter sp**.	—	—	—
	*Acinetobacter* sp.	—	—	—
	**Acinetobacter sp**.	—	—	—
	*Acinetobacter* sp.	—	—	—
	**Acinetobacter sp**.	—	—	*aadA27*
	**Acinetobacter sp**.	—	—	—
	*Acinetobacter* sp.	—	—	—
	**Acinetobacter sp**.	*bla*_MCA_	—	—
	*Acinetobacter guillouiae*	*bla*_OXA_	—	—
	**Enterococcus sp**.	—	—	—
	***Enterococcus aquimarinus***	—	—	*mef*(A)
	***Enterococcus aquimarinus***	—	—	—
	***Enterococcus aquimarinus***	—	—	—
	*Pseudomonas* sp.	—	—	—
	*Pseudomonas caeni*	—	—	—
Facility T	**Acinetobacter sp**.	—	—	—
	**Acinetobacter sp**.	*bla*_MCA_	*aph(6)‐Id, aph(3″)‐Ib*	*aadA27*
	**Acinetobacter sp**.	*tet*(39), *bla*_OXA_, *adeE*	—	—
	**Acinetobacter sp**.	—	—	—
	***Acinetobacter baumannii***	*bla*_ADC‐123_, *amvA*, *bla*_OXA‐65_, *ant(3″)‐IIa*	—	—
	*Acinetobacter baumannii*	ant*(3″)‐IIa*	—	—
	***Acinetobacter baumannii***	amvA, ant(3″)‐IIa, *bla*_ADC‐123_, *bla*_OXA‐65_	—	—
	***Acinetobacter brisouii***	—	—	—
	*Acinetobacter johnsonii*	*aac(3)‐I*	—	*bla*_MCA_
	*Acinetobacter johnsonii*	—	—	*bla*_MCA_
	*Acinetobacter parvus*	—	—	—
	***Acinetobacter pullicarnis***	—	—	—
	***Enterococcus aquimarinus***	—	—	*tet*(M)
	*Enterococcus aquimarinus*	—	—	—
	***Enterococcus aquimarinus***	—	—	—
	*Klebsiella* sp.	*bla*_ORN‐1_	—	*bla*_LEN_
	*Klebsiella ornithinolytica*	*oqxB*	—	—
	*Klebsiella pneumoniae*	*emrD*	—	*kdeA*, *oqxB*, *oqxA*
	***Pseudomonas boreopolis***	—	—	—
	**Pseudomonas sp**.	—	—	—

^a^ Bolded genus indicates the MAG was also found in the pool of filtered MAGs.

## 4 Discussion

Metagenomic sequencing has tremendous potential as a tool to enable the characterisation of genes encoding antibiotic resistance in complex microbial communities without cultivation. In this study, we considered the metagenomic DNA sequences in two distinctly different ways. The first method involved direct metagenomic sequencing assembled into contigs, similar to numerous previous studies (Che et al. 2019; Elbait et al. 2024; Guo et al. 2017; Wang et al. 2023; Zhang, Yang, and Pruden 2015). This approach generated results consistent with our expectations for the fate of ARGs during the municipal wastewater treatment process, thus providing a degree of validation for this dataset. Specifically, ARG levels were reduced from the untreated wastewater into the activated sludge and anaerobic digesters (Figure 1) and the composition of the ARGs clustered according to the location from which the sample was collected (Figure 2).

The second method also incorporated proximity‐ligation sequences, which were used to supplement short‐read shotgun metagenomic sequence data to generate more than 2000 medium‐quality MAGs (> 50% estimated genome completion, < 10% marker gene overrepresentation) from 21 samples collected from two full‐scale municipal wastewater treatment facilities. This enabled us to pair ARGs with a host MAG, even offering the ability to discern whether the ARG is located on the chromosome or on a plasmid within that MAG (Stalder et al. 2019). Although previous researchers have used long‐read metagenomic sequence data to associate ARGs with a host organism (Dai et al. 2022; Deshpande et al. 2023; Dong et al. 2024; Martin et al. 2021), the co‐location of potentially multiple ARGs within individual MAGs (and potentially metagenome assembled plasmid within a MAG) is a paradigm shift in our potential to understand the transition of the antibiotic resistome during municipal wastewater treatment.

This study demonstrates that untreated municipal wastewater contains numerous antibiotic resistant microbes, including those with ARGs on plasmids and ARGs hosted by ESKAPE‐like pathogens. Previous researchers have shown that untreated wastewater contains substantial concentrations of diverse ARGs via cultivation (Ramsden et al. 2010; Zhang et al. 2019), PCR (Buelow et al. 2018; Elbait et al. 2024; Leroy‐Freitas et al. 2022), and shotgun metagenomic sequencing (Che et al. 2019; Elbait et al. 2024; Rodríguez et al. 2021; Su et al. 2017). Cultivation is advantageous because it produces numerous bacterial isolates that can be analysed for resistance to multiple antibiotics and be subjected to genome sequence analysis; however, cultivation is well‐known to be biased to a small fraction of the bacterial community (Staley and Konopka 1985), such that the relevance of ARB isolated from untreated wastewater is questionable. PCR‐based approaches are highly sensitive and avoid the biases of cultivation, but these methods are also problematic because only intentionally targeted gene(s) can be analysed and the host of the target gene(s) remains unknown. Shotgun metagenomic sequencing can detect all known ARGs (i.e., the method is non‐targeted), but the sensitivity of this approach is limited by sequencing depth as well as the ability to accurately detect previously unidentified (Arango‐Argoty et al. 2018) and novel ARGs (Berglund et al. 2019). Our approach herein overcame many of these limitations by using the proximity‐ligation reads to link multiple contigs together to form more complete genomes that could be phylogenetically classified. For example, we identified numerous ESKAPE‐related organisms, many of which harboured ARGs, in untreated wastewater without cultivation. Of particular concern, multiple antibiotic‐resistant *Acinetobacter* spp. were detected in the untreated wastewater, consistent with the previous isolation of multiple antibiotic‐resistant *Acinetobacter* spp. from numerous locations (Kyriakidis et al. 2021; Rossolini et al. 2014). Perhaps most importantly, however, ESKAPE‐related organisms were not detected in either the activated sludge or the anaerobic digester samples, suggesting that municipal wastewater treatment effectively selects against these organisms of concern.

Our research also suggests that the prevalence of plasmid‐borne ARGs decreases from the untreated wastewater to either the activated sludge bioreactors or the anaerobic digesters. Plasmid‐borne ARGs are of particular concern because they are more susceptible to horizontal gene transfer (Brito 2021; Rodríguez‐Beltrán et al. 2021). Previous research has demonstrated that plasmids are easily detectable in activated sludge communities, as observed by the isolation of ARB that harbour plasmids (Fujita, Ike, and Suzuki 1993; Mach and Grimes 1982; McPherson and Gealt 1986), the direct extraction and purification of plasmids (Szczepanowski et al. 2009) and via the isolation of plasmids from conjugation experiments (Dröge, Pühler, and Selbitschka 2000; Schlüter et al. 2007). More recent research involving shotgun metagenomics has also detected numerous ARGs on contigs putatively identified as plasmids in wastewater treatment facilities, suggesting that activated sludge bioreactors have a lower prevalence of plasmid‐borne ARGs than the untreated wastewater (Che et al. 2019) and that anaerobic digesters have a lower relative abundance of plasmid‐borne ARGs than activated sludge bioreactors (Dai et al. 2022; Honda et al. 2023; Yoo et al. 2020) or the treated wastewater (Che et al. 2019; Szczepanowski et al. 2009). Our research, therefore, makes a novel contribution by also considering the relative abundance of plasmids and plasmid‐borne ARGs in anaerobic digesters treating municipal wastewater solids.

While the results from our proximity‐ligation metagenomic sequencing are mostly consistent with this prior work, our approach builds on this prior knowledge by pairing these putative plasmids contigs with specific MAGs and by often further utilising these contigs to create metagenome assembled plasmids. From a practical perspective, this additional information is critically important because the degree of concern associated with a plasmid‐borne ARG depends on its host; for example, a plasmid‐borne ARG is of more concern within an ESKAPE pathogen than within a *Nitrosomonas* spp. From an analytical perspective, this additional information confirms that some of the putative plasmid contigs are from genuine plasmids rather than chromosomal‐borne mobile genetic elements. However, because only a relatively small fraction (< 30%) of the ARGs found on putative plasmid contigs were assembled into metagenome assembled plasmids after incorporating the proximity‐ligation data, additional research is needed to better distinguish between genuine plasmid sequence information and chromosomal‐borne mobile genetic elements.

The primary goal of municipal wastewater treatment is to treat the water so that it can be returned to the environment without impacting surface water quality (Tchobanoglous et al. 2014). In addition, municipal wastewater facilities also could be designed to reduce and/or eliminate the antibiotic resistance in untreated wastewater. Prior research has demonstrated that approximately 95% of the antibiotic resistance in the untreated wastewater accumulates in the wastewater solids (Munir, Wong, and Xagoraraki 2011). One of the goals of this study was, therefore, to compare the ability of thermophilic anaerobic digestion and mesophilic anaerobic digestion to remove ARGs; our hypothesis is that higher operating temperatures would be more effective at reducing ARG levels compared to anaerobic digesters operated at approximately the temperature of the human body. Our research demonstrates that both thermophilic and mesophilic anaerobic digestion are effective at reducing the quantities of ARGs from the untreated wastewater, similar to previous studies (Diehl and LaPara 2010; Wang et al. 2024; Zhang, Yang, and Pruden 2015; Zhang et al. 2019). Curiously, this study suggested that thermophilic anaerobic digestion has statistically similar abilities to remove ARGs as mesophilic anaerobic digestion, which is similar to many previous studies (Ma et al. 2011; Zhang, Yang, and Pruden 2015; Huang et al. 2019) but contrasting other studies that suggested that thermophilic anaerobic digestion was superior to mesophilic anaerobic digestion at reducing the concentrations of ARGs in sewage sludge (Burch, Sadowsky, and LaPara 2016; Diehl and LaPara 2010; Ghosh, Ramsden, and LaPara 2009; Zhang et al. 2021).

Although proximity‐ligation metagenomic sequencing has enabled valuable insight into the transitions of the antibiotic resistome during municipal wastewater treatment, the technique has shortcomings that merit discussion. The most obvious limitation of our approach is that the assembly of MAGs (using shotgun and proximity‐ligation metagenomic sequences) is a computational prediction of genuine genomic sequences; the validity of these assemblies from communities as complex as those in untreated wastewater, activated sludge bioreactors, and anaerobic digesters is not known. Another pertinent limitation of this approach is that the MAGs generated represent only the most prominent microorganisms in these complex and dense microbial communities; prior research has demonstrated, for example, that numerous ARGs are easily detectable by quantitative PCR at levels well below that capable by metagenomic sequence analysis (Diehl and LaPara 2010; Elbait et al. 2024; LaPara et al. 2011; Munir, Wong, and Xagoraraki 2011). This limitation is particularly pertinent when considering our conclusion that municipal wastewater treatment selects against ESKAPE‐related organisms (especially those harbouring ARGs). That is, our results demonstrated that ESKAPE‐related organisms were not detectable in the activated sludge bioreactors and anaerobic digesters at either municipal wastewater treatment facilities, but these organisms were still likely present within these samples, albeit at low concentrations that could not be detected by metagenomic sequencing. Our research also suggests that proximity‐ligation metagenomic sequencing dampens quantitative differences in the prevalence of ARGs within a microbial community profile. Specifically, statistically significant differences were often observed in the contigs obtained via direct metagenomic sequencing that were not observed when comparing ARGs in the pools of MAGs or filtered MAGs (Figures 1, 2, 3).

In conclusion, our research demonstrates that the antibiotic resistome makes significant transitions in the quantity, composition, and hosts during the municipal wastewater treatment process. Untreated municipal wastewater contained higher concentrations of ARGs, ARGs located on plasmids and ESKAPE‐related organisms (including ESKAPE‐related organisms harbouring plasmid‐borne ARGs) than either the activated sludge bioreactors or anaerobic digesters. In addition, the organisms harbouring ARGs in the untreated wastewater, activated sludge bioreactors and anaerobic digesters were specific to those unit operations. These novel scientific contributions were enabled by proximity‐ligation metagenomic sequencing, which allowed us to pair ARGs and plasmids with their putative microbial hosts (i.e., MAGs).

## Author Contributions

**Cassandra B. McCorison:** data curation, formal analysis, software, methodology, visualization, writing – original draft. **Taegyu Kim:** formal analysis. **Justin J. Donato:** conceptualization, funding acquisition, project administration, supervision, writing – review and editing. **Timothy M. LaPara:** conceptualization, funding acquisition, project administration, writing – original draft, supervision.

## Conflicts of Interest

The authors declare no conflicts of interest.

## Supporting information


Figure S1.
Figure S2.
Figure S3.

## Data Availability

The data that support the findings of this study are openly available in NCBI at https://www.ncbi.nlm.nih.gov/genbank/, reference number PRJNA1129166.
