# Supplementary material for: Proximity‐Ligation Metagenomic Sequence Analysis Reveals That the Antibiotic Resistome Makes Significant Transitions During Municipal Wastewater Treatment
Source: Environ Microbiol. 2025 Jan 10;27(1):e70036. doi: 10.1111/1462-2920.70036 (PMC11724201; doi:10.1111/1462-2920.70036)
Supplement: Supplementary file 1 — Figure S1. Figure S2. Figure S3. [file EMI-27-e70036-s001.docx]

**SUPPORTING FIGURES**


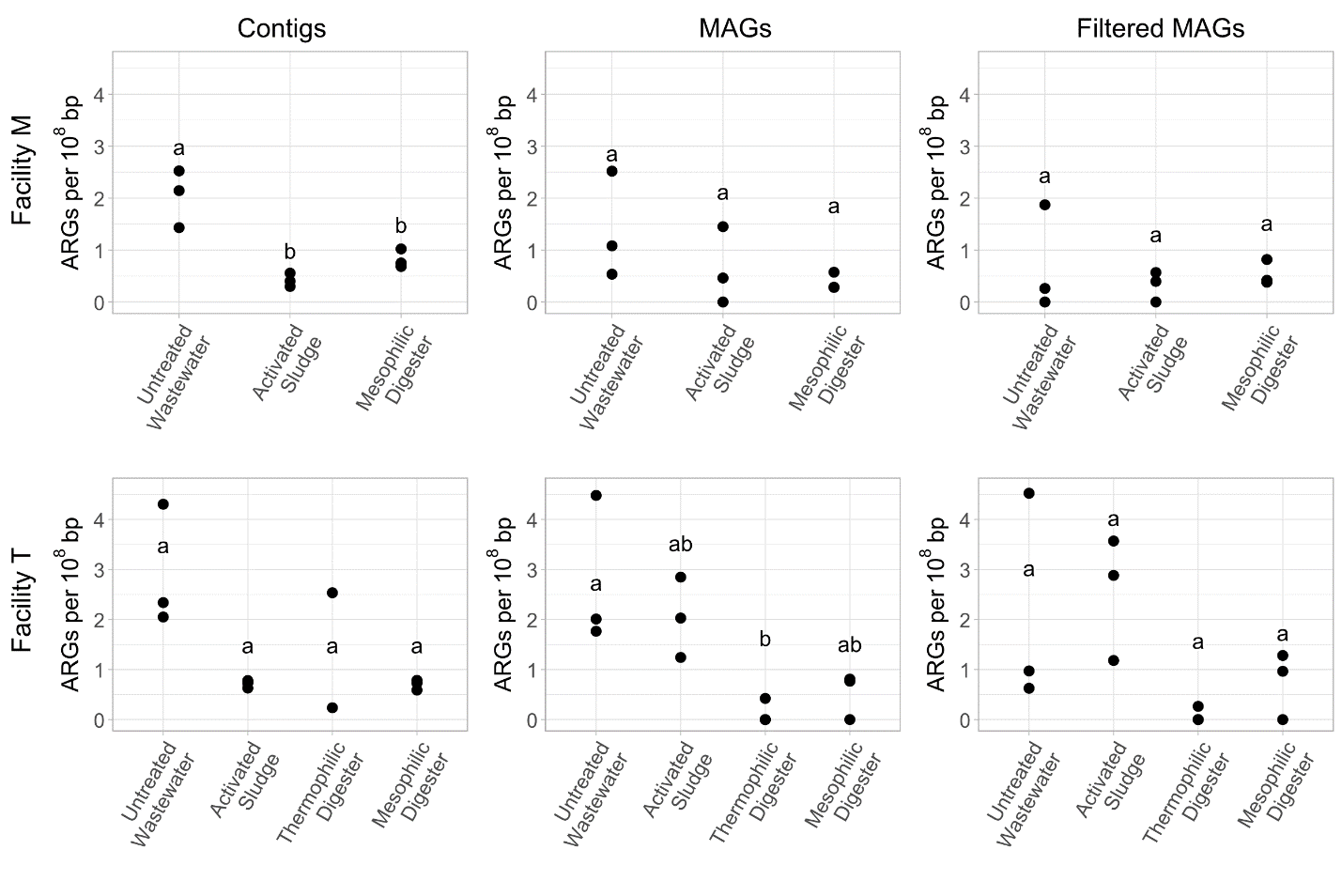


**Figure S1.** The relative abundance of genes encoding resistance to macrolides in the untreated wastewater, activated sludge bioreactors, and anaerobic digesters from two full-scale municipal wastewater treatment facilities (Facility M: top panel; Facility T: bottom panel). Results were computed from metagenomic sequences directly assembled into contigs, from proximity ligation sequence results assembled into metagenome assembled genomes (MAGs), and from the pool of “filtered” MAGs, which excluded MAGs for insufficient completeness (< 50%) or for excessively high marker gene overrepresentation rate (> 10 %).


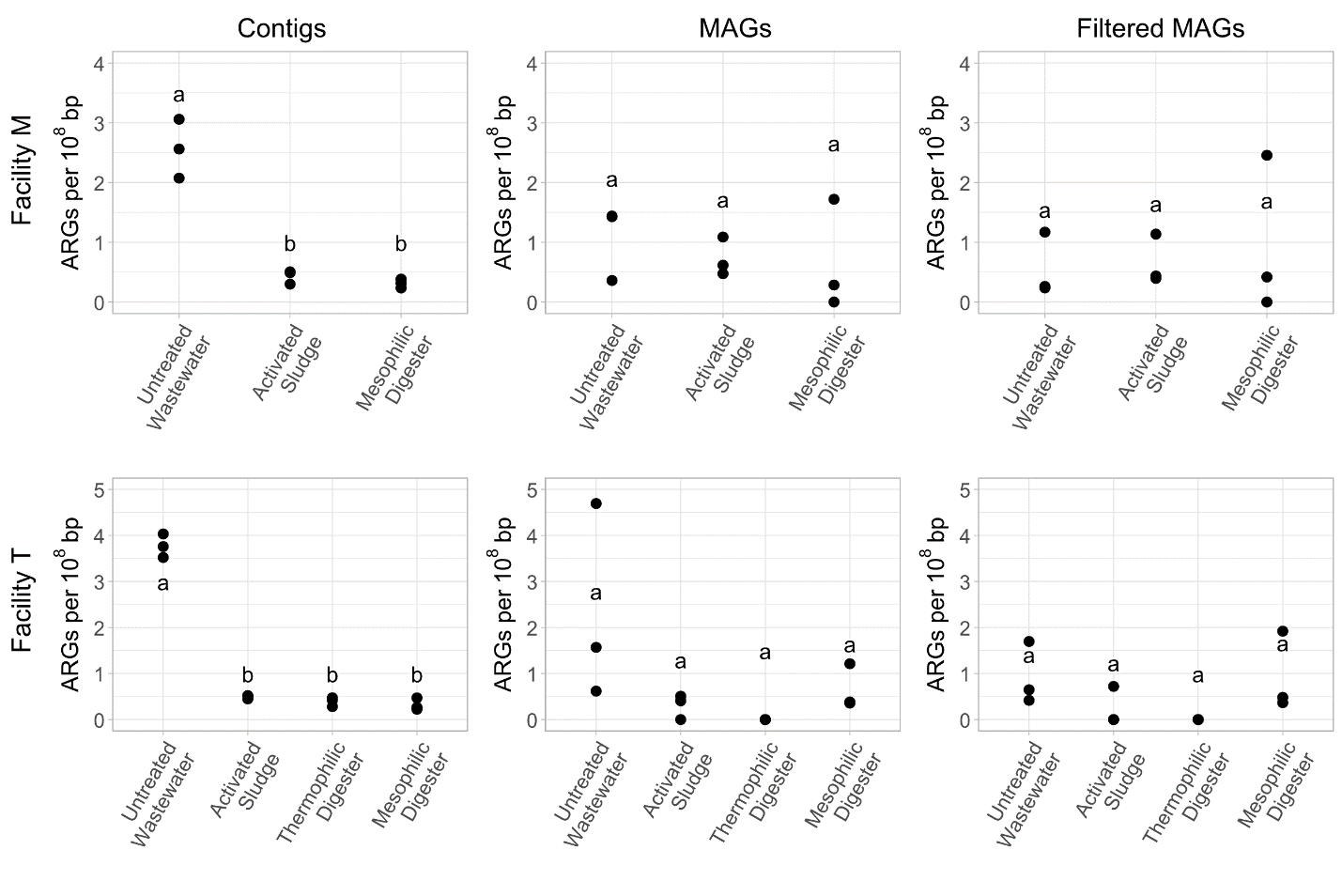


**Figure S2.** The relative abundance of genes encoding resistance to β-lactams in the untreated wastewater, activated sludge bioreactors, and anaerobic digesters from two full-scale municipal wastewater treatment facilities (Facility M: top panel; Facility T: bottom panel). Results were computed from metagenomic sequences directly assembled into contigs, from proximity ligation sequence results assembled into metagenome assembled genomes (MAGs), and from the pool of “filtered” MAGs, which excluded MAGs for insufficient completeness (< 50%) or for excessively high marker gene overrepresentation rate (> 10 %).


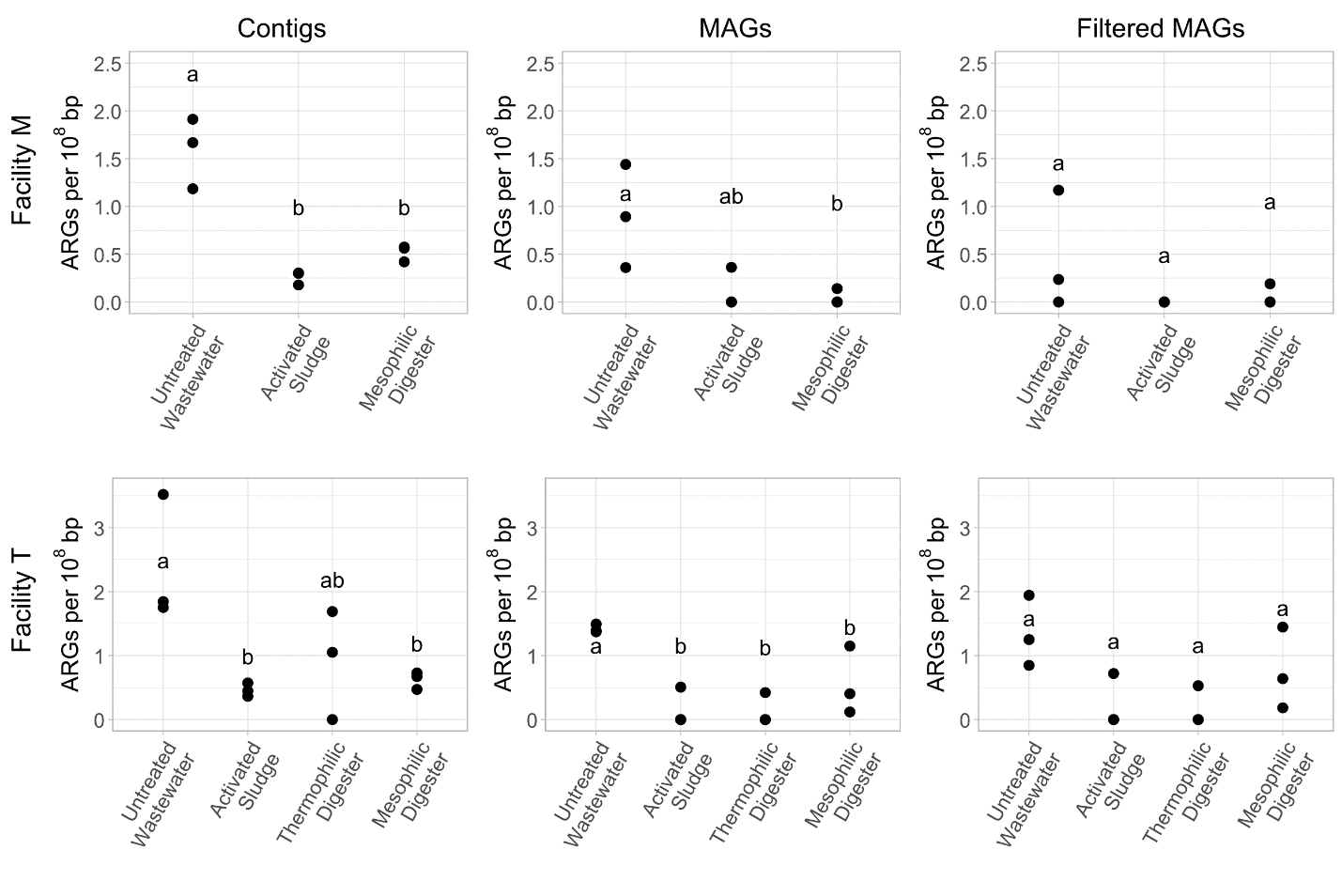


**Figure S3.** The relative abundance of genes encoding resistance to tetracyclines in the untreated wastewater, activated sludge bioreactors, and anaerobic digesters from two full-scale municipal wastewater treatment facilities (Facility M: top panel; Facility T: bottom panel). Results were computed from metagenomic sequences directly assembled into contigs, from proximity ligation sequence results assembled into metagenome assembled genomes (MAGs), and from the pool of “filtered” MAGs, which excluded MAGs for insufficient completeness (< 50%) or for excessively high marker gene overrepresentation rate (> 10 %).
